# Advanced Detection of Pancreatic Cancer Circulating Tumor Cells Using Biomarkers and Magnetic Particle Spectroscopy

**DOI:** 10.7150/ntno.110074

**Published:** 2025-06-12

**Authors:** Ali Dinari, Hafiz Ashfaq Ahmad, Seungjun Oh, Yun-Hee Kim, Jungwon Yoon

**Affiliations:** 1 Department of AI Convergence, Gwangju Institute of Science and Technology, Gwangju, 61005, Republic of Korea.; 2 Department of Medical Physics, M. Smoluchowski Institute of Physics, Jagiellonian University, ul. S. Łojasiewicza 11, 30-348, Krakow, Poland.; 3 National Cancer Center, Goyang-si, Gyeonggi-do, 10408, Republic of Korea.

**Keywords:** nanotheranostic platforms, circulating tumor cells (CTCs), pancreatic cancer

## Abstract

**Background:** Pancreatic ductal adenocarcinoma (PDAC) typically develops without symptoms, and its aggressive progression combined with late-stage diagnosis underscores the critical need for improved early detection strategies. Circulating tumor cells (CTCs) in blood are potential biomarkers for PDAC. In this study, the detection of pancreatic cancer-associated CTCs was evaluated using two magnetic-based diagnostic systems in a comparative approach.

**Methods:** Two distinct nanotheranostic platforms were developed: monoclonal antibody-conjugated magnetic nanoparticles (mAbs-MNPs) and magnetized exosomes (termed Magxosomes). Anti-mesothelin and anti-vimentin were used as monoclonal antibodies, while mesenchymal stem cells (MSCs) treated with MNPs served as the source of Magxosomes. Characterization of nano-systems was performed using dynamic light scattering (DLS), inductively coupled plasma mass spectrometry (ICP-MS), nanoparticle tracking analysis (NTA), and transmission electron microscopy (TEM). Blood samples were collected from pancreatic cancer mouse models, treated with nanotheranostic platforms, and analyzed using a homemade magnetic particle spectroscopy (MPS) device.

**Results:** The detection of pancreatic cancer-associated CTCs was investigated using nanotheranostic platforms alongside an MPS instrument. In this context, the mAbs-MNPs systems demonstrated varying efficiencies in the diagnosis of CTCs, with Ant-V-MNPs (anti-vimentin conjugated MNPs) achieving 27.47%, Ant-M-MNPs (anti-mesothelin conjugated MNPs) at 13.59%, and a 50:50 mixture of Ant-M-MNPs: Ant-V-MNPs showing an efficiency of 19.73%. Conversely, the efficiencies of Magxosomes were notably higher. Bone marrow stem cell (BMSC)-derived Magxosomes achieved an average efficiency of 63.39%, while adipose-derived stem cell (ADSC)-derived Magxosomes exhibited an average efficiency of 56.23%.

**Conclusions:** This study introduces a promising method for early pancreatic cancer diagnosis by detecting CTCs in blood. It employs a non-invasive, rapid test using an advanced MPS instrument (1 ng detection limit) and nanotheranostic platforms. Results confirm the system's robustness in identifying pancreatic cancer CTCs. This approach may support future developments in cancer diagnostics and monitoring.

## Introduction

Pancreatic ductal adenocarcinoma (PDAC) is classified as a highly fatal cancer 1. Prospective studies show that it will be the second leading cause of cancer-related deaths by the year 2030. It is often diagnosed at metastatic stages, which reflects the low survival rate 2. Circulating tumor cells (CTCs), with the ability to detach from the primary tumor, act as the seeds for cancer metastasis. They undergo a process called epithelial-mesenchymal transition (EMT), during which their morphology changes 3. The EMT process results in a continuum of epithelial to mesenchymal phenotypes with variations in their expressed proteins, creating a heterogeneous pool of tumor cells 4-6. CTCs are promising candidates for cancer diagnosis, therapeutic monitoring, and relapse identification 7. However, the numbers of CTCs in peripheral blood are inadequate. To enrich and isolate CTCs, several technologies have been developed. Variations in size, density, deformability, and electrical properties between CTCs and blood cells are crucial parameters for physical separation. Low efficiency and a large volume of required blood limit this method 8, 9. Biological properties-based isolation systems have been developed and rely on antibody-antigen interactions. In this regard, the Cell Search system was developed based on Ferromagnetic coated epithelial cell adhesion molecule (EpCAM). However, the adhesion of CTCs during antibody interactions and heterogeneity of CTC surface antigens are disadvantages of this method 10, 11. The detection of CTCs based on biomarkers represents several advantages. Mesothelin, a cell-surface glycoprotein with normal expression limited to mesothelial cells, showed a high level of expression in many cancers including PDAC, lung adenocarcinoma, ovarian cancer, etc. 12-14. The other promising candidate is vimentin, necessary to maintain the cellular integrity of mesenchymal cells 15. Evidences confirmed that vimentin is overexpressed in various epithelial cancerous cells (PANC-1 and MIA PaCa-2 in pancreatic cancer) 16, 17. It has the potential to identify mesenchymal phenotypes of CTCs 18. Extracellular vesicles (EVs) are another bioactive structure with remarkable capacity in diagnosis and therapy. Exosomes, a subset of EVs with an average size between 30-200 nm, play a crucial role in intercellular communication and are implicated in disease pathogenesis 19, 20. They reflect the content of the cell of origin and indicate alterations in their status. Clinical applications of exosomes are extended in diagnostic and therapeutic research areas 21.

On the other hand, magnetic nanoparticles (MNPs) are well-known for nanomedicine application 22, 23. The Features of functionalization and guidance by magnetic field enable them to be versatile tools for guided tracking and delivery systems. By manipulating MNPs with biological agents, they can overcome the cellular and tissue barriers while maintaining their therapeutic and diagnostic features 24. Steering MNPs using an external magnetic field offers molecular tracking via magnetic resonance imaging (MRI) and magnetic particle imaging (MPI) 22. Indeed, MPI is a cutting-edge technology that enables the quantification and local tracking of MNPs at desired regions by directly scanning them as an imaging tracer 25, 26. The zero-dimensional MPI termed as Magnetic particle spectroscopy (MPS), is a new modality for detecting the signals of MNPs regardless of their location. Both modalities of MPS and MPI are based on the same nonlinear dynamic properties of MNPs 27, 28. In company with MNPs and biomarkers, the MPS modality provides a rapid, sensitive, and versatile platform for the diagnosis of pancreatic cancer-related CTCs. However, CTCs involve many hindrances. They are extremely rare in blood fluid, exhibit heterogeneity in both phenotypes and genotypes, can form clusters, and lodge in capillaries. In addition, their dissemination in blood circulation varies both spatially and temporally due to partial filtration 29-31. Nowadays, research studies are trying to combine the specificity of tumor markers with advanced technology-based modalities to increase the accuracy of detection. Applying dual-antibody-modified fluorescent MNPs or antifouling hydrogel-coated MNPs to isolate blood-based CTCs are remarkable examples 32, 33.

This research aimed to develop a novel method for the detection of pancreatic cancer-related CTCs. To this end, the desired nanotheranostic platforms were armed, optimized, and characterized separately. These include monoclonal antibody-conjugated magnetic nanoparticles (mAbs-MNPs), such as anti-mesothelin-conjugated MNPs (Ant-M-MNPs), anti-vimentin-conjugated MNPs (Ant-V-MNPs), and magnetized exosomes (Magxosomes). Subsequently, the validated systems were individually added to blood samples and investigated using an advanced home-built MPS device to diagnose pancreatic cancer-related CTCs. The diagnostic capability of mAbs-MNPs and Magxosomes platforms for CTC detection was evaluated using blood samples from a pancreatic cancer model mouse. The obtained results were indicative of the superiority of the designed approach in CTCs detection. It seems sufficiently innovative for the early diagnosis of pancreatic cancer.

## Materials and Methods

Florescent magnetic nanoparticles (Cat No: 125-01-501) were purchased from Micromod Company (Rostock, Germany). The chemicals such as 1-Ethyl-3-(3-dimethylaminopropyl) carbodiimide (EDC, CAS No: 25952-53-8), N-Hydroxysuccinimide (NHS, CAS No :6066-82-6), MES (2-(N-Morpholino) ethanesulfonic acid hydrate, 4-Morpholineethanesulfonic acid, CAS No: 4432-31-9) were purchased from Sigma-Aldrich Inc, (South Korea). Monoclonal antibodies including anti-mesothelin (Cat No: 13128-MM01), and anti-vimentin (Cat No: 100254-R001) were purchased from Sino Biological Company (China). Mesenchymal stem cells (MSC) including adipose-derived stem cells (ADSC) and bone marrow stem cells (BMSC) were purchased from iXCells Biotechnologies Company (USA).

MSC-specific cell mediums (Cat No: MD-0003b and MD-0037) were purchased from iXCells Biotechnologies company (USA). Falcon multi-flask (3-layer tissue culture-treated, 525 cm^2^, Cat No:353143), and Total exosome isolation kit (Cat No: 4478359, Invitrogen) were purchased from Thermo Fisher Scientific (USA). BALB/c 6 weeks' female nude mouse CFPAC-1 subcutaneous model as the pancreatic model mouse were prepared by National Cancer Center of Korea (Seoul, South Korea). The chemicals include 0.5M EDTA pH 8.0 (Cat No: MEDTA 001), Citrate-phosphate-dextrose solution (CPD, LOT No: SLBP3291V) were prepared by Sigma Aldrich (South Korea).

MPS instrument: The MPS device used for capturing MNP signals was a homemade designed and fabricated in the Intelligent Medical Robotics Lab, (GIST, South Korea). It has optimized excitation coils and highly sensitive receiver coils to capture the emitted signals from MNPs. A small AC excitation field of 2 mT at 24.36 kHz frequency was applied through a power amplifier (AE Techron 7724), and a bandpass excitation filter was used to ensure the linear amplification of one frequency signal to excite the MNPs in the sample. Due to the non-linear behavior of the MNPs, a signal is generated in the receive channel, which is further passed through a Lock-In amplifier (SR865A 4 MHz DSP Lock-in Amplifier) to measure the third harmonic of the sample's signal as the output. Amplification and filtration steps further enhanced signal quality by mitigating unwanted noise and interference. The signal is then processed through NI PXIe-7867 and PCIe-6363 (National Instruments, Austin, TX). The system could detect MNPs at concentrations of 10 ng, 7 ng, and 1 ng, with 10 ng detected in 5 seconds (integrated signal) and 1 ng in 20 seconds. This demonstrates the system's potential for sensitive measurements in biological applications.

Conjugation process: To this end, the EDC-NHS conjugation protocol was applied to attach monoclonal antibodies (mAbs) to the surfaces of MNPs (5 mg/ml) 34-37. In brief, anti-mesothelin (14.7 µM) and anti-vimentin (18.18 µM) monoclonal antibodies (mAbs) were added to individual tubes (1.5 ml) containing plain MNPs. The ratio between MNPs and proteins was 1:5 respectively. To keep this ratio fixed, the applied concentration of MNPs per mAbs was 5 mg/ml / 0.44 µM. The conjugation process was performed in MES buffer with the pH adjusted to 6. The reaction agents, including MES buffer, MNPs, mAbs, and EDC-NHS powders, were added to the reaction tube, pipetted twice at a 15-minute interval, and shaken at 50 RPM for 2 hours. Next, the reaction tubes were placed on magnetic beads for 24 hours and then washed with PBS to separate the conjugated proteins from the unreacted ones. This step was repeated three times to ensure the removal of unreacted proteins.

Cell culture experiments: Cell culture experiments were conducted with mouse-specific MSC including ADSC and BMSC. At the desired confluency (80%), the Ant-V-MNPs were added to the cell medium. To attain the highest number of treated cells, they were proliferated in the falcon multi-flask. Subsequently, the cells were harvested by accutase treatment and centrifugation, sub-cultured with fresh medium (depleted vesicles FBS), and incubated for 48 hours. In the end, the cell medium was isolated as the source of Magxosomes. It is mentioned that depleted vesicle FBS was prepared using ultracentrifugation at 100,000g for 18 hours at 4°C. This process was performed before adding it to the cell culture medium. The process was necessary to ensure that isolated Magxosomes were obtained from MSC.

Magxosomes isolation: For the isolation of Magxosomes, the MSC medium was treated with an adequate volume of exosome extraction kit solvent. In brief, the MSC-isolated medium was centrifuged at 2000g for 30 minutes to remove the cell's debris. The obtained cell-free supernatant was transferred to a new tube and mixed with the kit reagent solvent according to the standard protocol. The reaction was completely homogenous through vortex (or pipette). Next, the homogenous mixture was incubated at the refrigerator (2°- 8°C) overnight. After incubation, the samples were centrifuged (10,000g, 1 h, 2°C) and discarded the supernatant. The accumulated pellet of exosomes at the bottom of the tube (often not visible) was re-suspended in a convenient volume of PBS and kept at -80°C for long-term storage.

Blood collection: The BALB/c-nu mice were used to establish the pancreatic cancer model. For tumor implantation, the pancreatic cancer cell line CFPAC-1 was obtained from the American Type Culture Collection (ATCC) and cultured in Iscove's Modified Dulbecco's Medium (IMDM; Gibco), supplemented with 10% (v/v) FBS and 100 IU/mL antibiotic-antimycotic solution (Gibco). Six-week-old BALB/c-nu mice were subcutaneously injected in the flank with CFPAC-1 cells (5 × 10⁶ cells in 100 μL PBS) to establish a pancreatic cancer model. After two weeks (i.e., at eight weeks of age), body weight and tumor size were carefully measured, after which the mice were euthanized for blood collection. Following euthanasia, tumor weight was also recorded. All animal procedures were reviewed and approved by the Institutional Animal Care and Use Committee (IACUC) of the Gwangju Institute of Science and Technology (GIST) (IACUC approval number: GIST-2021-089, GIST-2021-051). Anesthesia was administered according to the standard protocol for deep inhalation anesthesia to ensure the mice were unconscious and completely insensitive to pain during blood collection. A total of 36 mice were sacrificed, and blood was extracted from the posterior vena cava. In general, this method of blood sampling is recommended for the terminal stage of the study. The collected volume of blood was almost between 500 - 700 µl. To prevent blood coagulation, the syringe's needle (25G) was embedded in 0.5M EDTA solvent and pipetted to deplete the bubbles. The collected samples of blood transferred to tubs contain the CPD solvent. The ratio of blood to CPD was 1:7, according to the manufacturer's instructions. It is mentioned that the blood can be preserved in the refrigerator for 21 days after mixing with CPD buffer.

Treatment of blood samples with nanotheranostic platforms: The collected blood samples were treated with mAbs-MNPs and Magxosomes systems separately. In the experimental design, the treatments included PBS, untreated blood, and blood treated with Ant-V-MNPs, Ant-M-MNPs, Ant-M-MNPs:Ant-V-MNPs (50:50), BMSC-derived, and ADSC-derived Magxosomes (supplementary information Table S4). The number of sacrificed mice was 12 per experiment. Two blood samples were dedicated to each treatment (all treatments except PBS). The volume ratio of blood to mAbs-MNPs and Magxosomes was 450 µl to 50 µl, respectively, with concentrations of 2 mg/ml for mAbs-MNPs and 0.02 mg/ml for Magxosomes. In the next, the treated samples were shaken for 1 hour, mixed with the same volume of PBS, pipetted very gently. Finally, the samples were centrifuged (2500 rpm for 7 minutes) and washed three times with clinical-grade PBS. To maintain the integrity of the blood cells, the centrifuge speed was kept below 2500 rpm.

Characterization tests: Characterization of mAbs-MNPs and Magxosomes systems performed through the instruments of dynamic light scattering (DLS, Malvern, UK), nanoparticle tracking analysis (NTA, NanoSight LM10 system, Malvern, UK), transmission electron microscopy (TEM, 300 kHz, Tecnai G2 F30 S-Twin FEI, USA) and inductively coupled plasma mass spectrometry (ICP-MS (7900, Agilent, Japan). It is noteworthy that the above-mentioned instruments have been used with respect to their relative standard developed protocols.

## Results and Discussion

**Figure 1:**
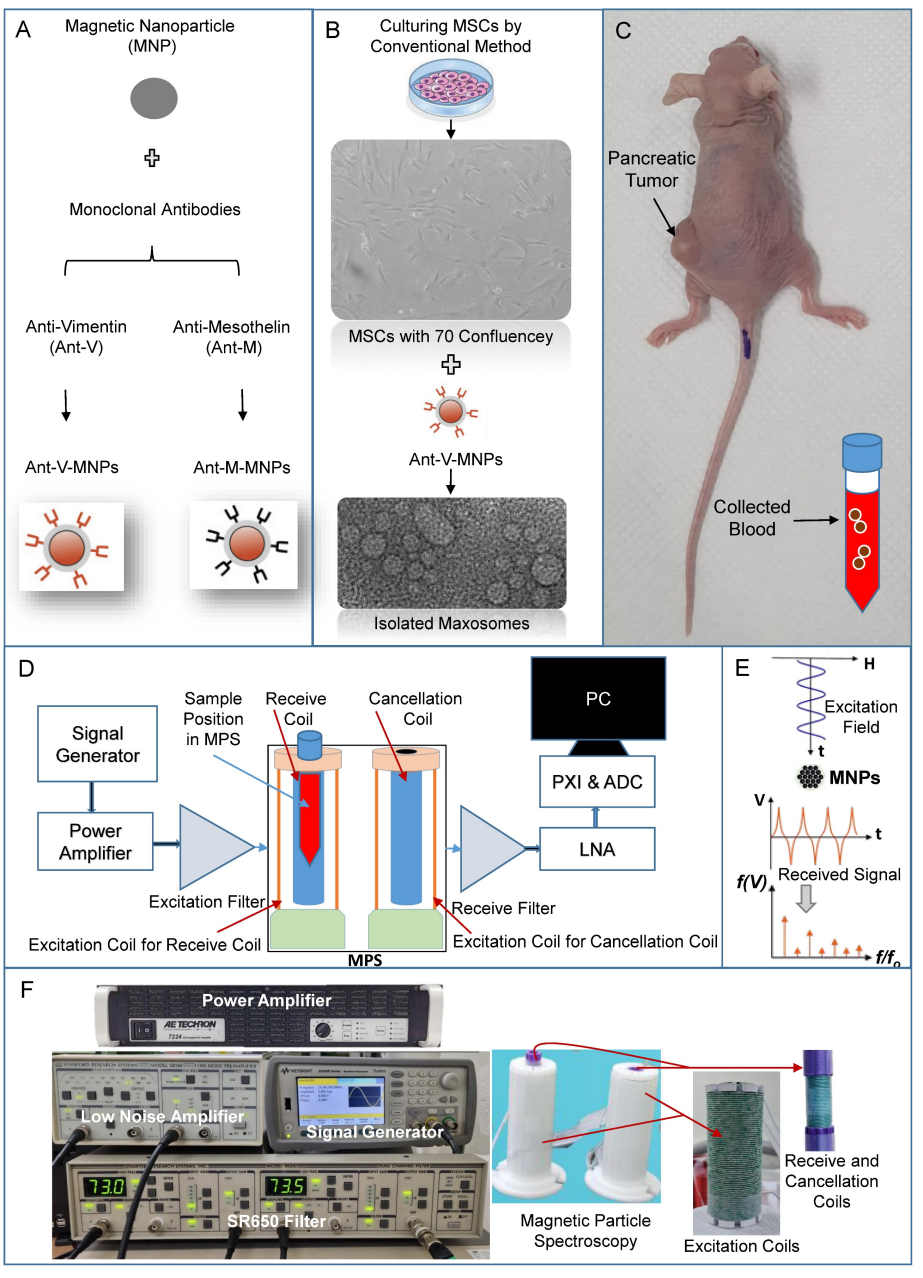
The representation includes the preparation of applied nanoplatforms (mAbs-MNPs and Magxosomes), blood sampling from a pancreatic mouse model (MNPs), and the use of a homemade device for measuring magnetic signals. (A) mAbs-MNPs fabricated from conjugation of monoclonal antibodies (Anti-Vimentin and Anti-Mesothelin) to the MNPs surfaces. (B) isolation of Magxosomes from mesenchymal stem cells. (C) A pancreatic tumor mouse model was used as the source of collected blood. (D) schematic of designed MPS, (E) represents the excitation of MNPs and received signal in time domain and in frequency domain, and (F) represents designed hardware, excitation and receive coils of MPS, low noise amplifier (LNA), signal generator, power amplifier and SR650 which is used as a band-pass filter to record only the 3^rd^ harmonic.

High rate of metastasis occurrence and vascular invasion lead to the lethality condition of pancreatic cancer 38. The CTCs are malignant cells that dissociate from the primary tumor, enter the bloodstream, travel to the distant tissues, extravasate, adapt to the new microenvironment, and colonize to form metastases 39. Detection of CTCs in the bloodstream can provide valuable information regarding the status of cancer disease. However, the camouflage ability of CTCs and their small quantity in the blood are the parameters that make the CTCs tracking challenging 40. To achieve accurate therapy, a precise diagnosis is crucial. The evaluation of disease stages using the most accurate method is fundamental for choosing the best therapeutic strategy 41. The ability to trace the MNPs via MPI as the new technology for the detection of MNPs, distinct from biological milieu effects, is a new approach in molecular imaging. MPS, as the zero-dimensional MPI, can detect and quantify very low, sub-nanomolar concentrations of molecular biomarkers regardless of the location of MNPs 42. Biomarkers are always crucial because of their roles as measurable indicators. Besides many applicable features, they can be targeted for cargo delivery and imaging 43. The biomarkers, such as mesothelin and vimentin proteins or exosome cargos can cover the most features that are essential for suitably applied biomarkers for purposes of diagnosis and molecular imaging. Taken together, the advantages of MNPs, biomarkers, and MPS modality can be combined in an intelligent diagnostic system to pursue pancreatic cancer-related CTCs. In this research, two distinct nanotheranostic platforms were used, and their ability to track pancreatic cancer-related CTCs was investigated using an advanced, homemade MPS device (Figure 1). The obtained results indicated that this method could be a promising and non-invasive way to determine disease progression.

### Characterization of nanotheranostic platforms and CTC diagnosis

DLS analysis: Measurement of size and zeta potential of mAbs-MNPs samples using the DLS instrument confirmed that the process of conjugation had occurred correctly. The plain MNPs and conjugated mAbs-MNPs (Ant-M-MNPs and Ant-V-MNPs) showed different values for size and zeta potential. Related to the size, it was illustrated that due to the conjugation of protein on the surface of MNPs, the hydrodynamic size of Abs-MNPs was increased. In this regard, the average size of Ant-V-MNPs was approximately 68.68 nm, while that of Ant-M-MNPs was 61.86 nm, compared to plain MNPs with an average size of 53.18 nm. The structures of vimentin and mesothelin differ significantly in shape, which influences the final hydrodynamic size of the conjugated MNPs. Mesothelin, a membrane-bound glycoprotein with a molecular weight of 40 kDa, has a compact, globular structure on the surface of mesothelial cells. In contrast, vimentin, with a molecular weight of 53.7 kDa, forms long, fibrous filaments within the cytoplasm and is characterized by a helical coil structure 44-46. Consequently, the size of Ant-V-MNPs would be about 6.82 nm larger than that of Ant-M-MNPs. The recorded data approved that adding the protein to the surface of MNPs resulted an increase in size, so the average size of plain MNPs, Ant-M-MNPs, and Ant-V-MNPs were 53.18, 61.9, and 68.7 nm respectively.

**Figure 2:**
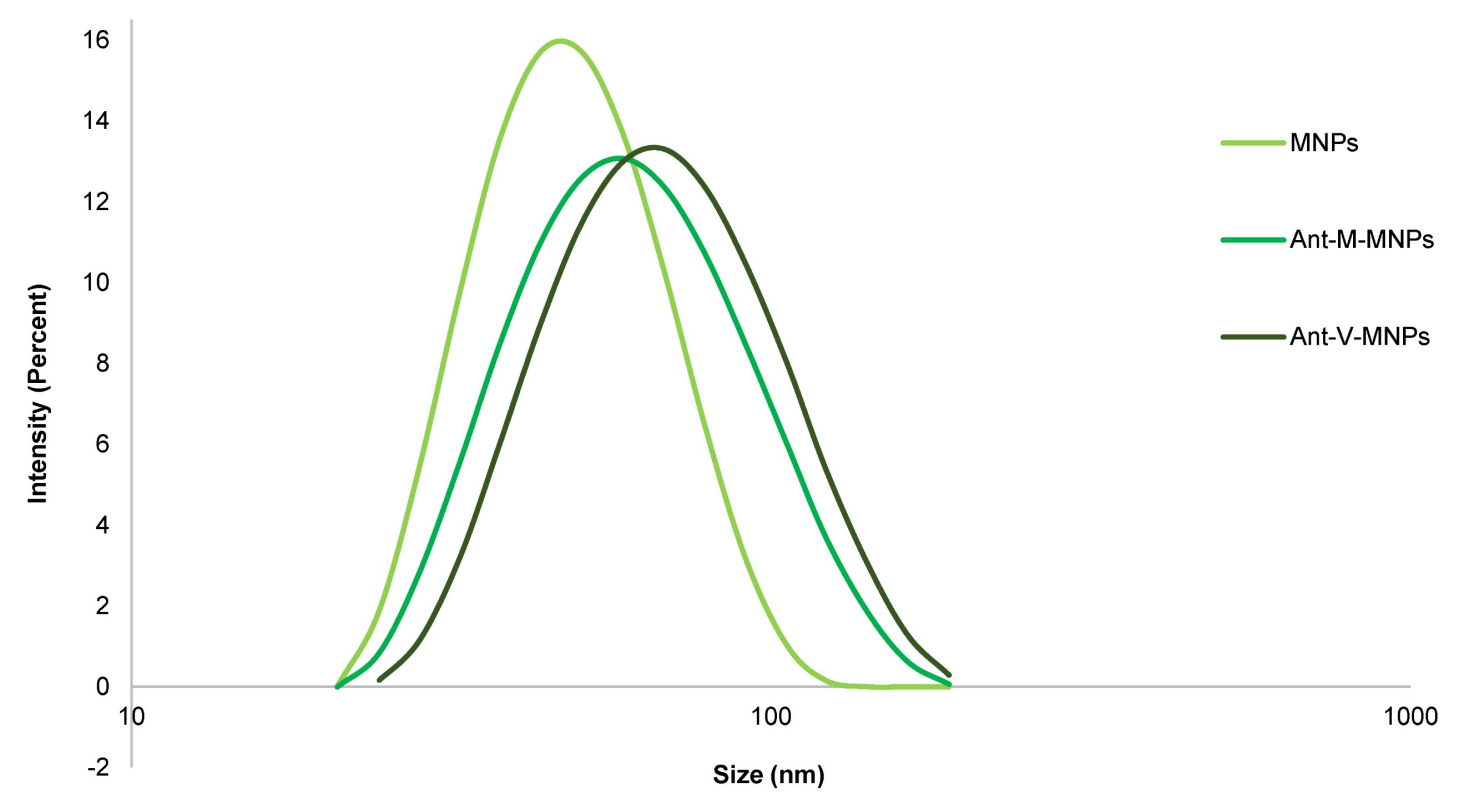
Size distribution of different MNPs samples. The plot compares the hydrodynamic size distribution of MNPs and conjugated MNPs (Ant-M-MNPs and Ant-V-MNPs).

The zeta potential parameter could also provide evidence to approve the accuracy of conjugation. In this regard, recorded data were (11.33 mV), (-5.37 mV), and (-5.17 mV) for MNPs, Ant-M-MNPs, and Ant-V-MNPs respectively. Shifting from positive values towards negative advocates the conjugation accuracy and addition of proteins on MNPs surfaces. The zeta potential of dextran is generally neutral. However, surface modifications, such as the introduction of carboxyl, sulfate, or amino groups, can lead to either negative or positive zeta potentials. In this research, dextran functionalized with amino groups exhibits a positive zeta potential. Upon conjugation, a portion of the amino groups interacts with the carboxyl (-COOH) groups of the protein, reducing the overall charge of the conjugated MNPs 47, 48. The Polydispersity index (PDI) which indicated the heterogeneity and distribution of sample sizes, also confirmed the effect of connected proteins on the MNPs surface. The above-mentioned measured parameters are shown by Figure 2 and Table 1.

It is noteworthy that, to improve the conjugation process between MNPs and antibodies (anti-mesothelin and anti-vimentin) and reduce the number of non-conjugated MNPs, it can be optimized though the following approaches: One key strategy for enhancing conjugation yields is improving conjugation efficiency by optimizing the molar ratio of protein to MNPs in fine-tuning reaction conditions such as pH, temperature, and incubation time. Detailed protocols for such optimizations are well-documented in the literature 34. The protocol applied in the present study was strictly aligned with the principles established in prior research. Another effective approach involves surface functionalization of MNPs. Uniform and dense coatings of functional groups such as carboxyl or amine groups can significantly increase coupling efficiency and reduce the presence of unbound particles 49. The Synomag MNPs, used in this research were coated with amine groups, which enhanced the coupling efficiency and facilitated the conjugation process. From a technical standpoint, the conjugation process can be further improved by employing stepwise and gentle washing protocols. Using mild magnetic separation techniques in combination with optimized PBS volumes and minimizing the number of wash cycles can help reduce mechanical stress and prevent particle loss 50. In the present research, three washing steps with standard PBS were sufficient to prevent mechanical stress and minimize particle loss. Moreover, alternative separation techniques, such as size-exclusion chromatography or ultrafiltration, may be employed to remove unbound MNPs without repeated magnetic separation steps, thereby preserving conjugation efficiency 51. Further optimization can be achieved by incorporating pre-blocking agents such as BSA or PEG to reduce aggregation and enhance the stability of MNP conjugates 52. Quantitative monitoring of conjugation efficiency is also crucial; techniques like ICP-MS for iron quantification and UV-Vis spectroscopy for protein content can provide valuable insights into yield and losses 53. Implementing these strategies is expected to substantially enhance the overall efficiency of MNP conjugation.

**Figure 3:**
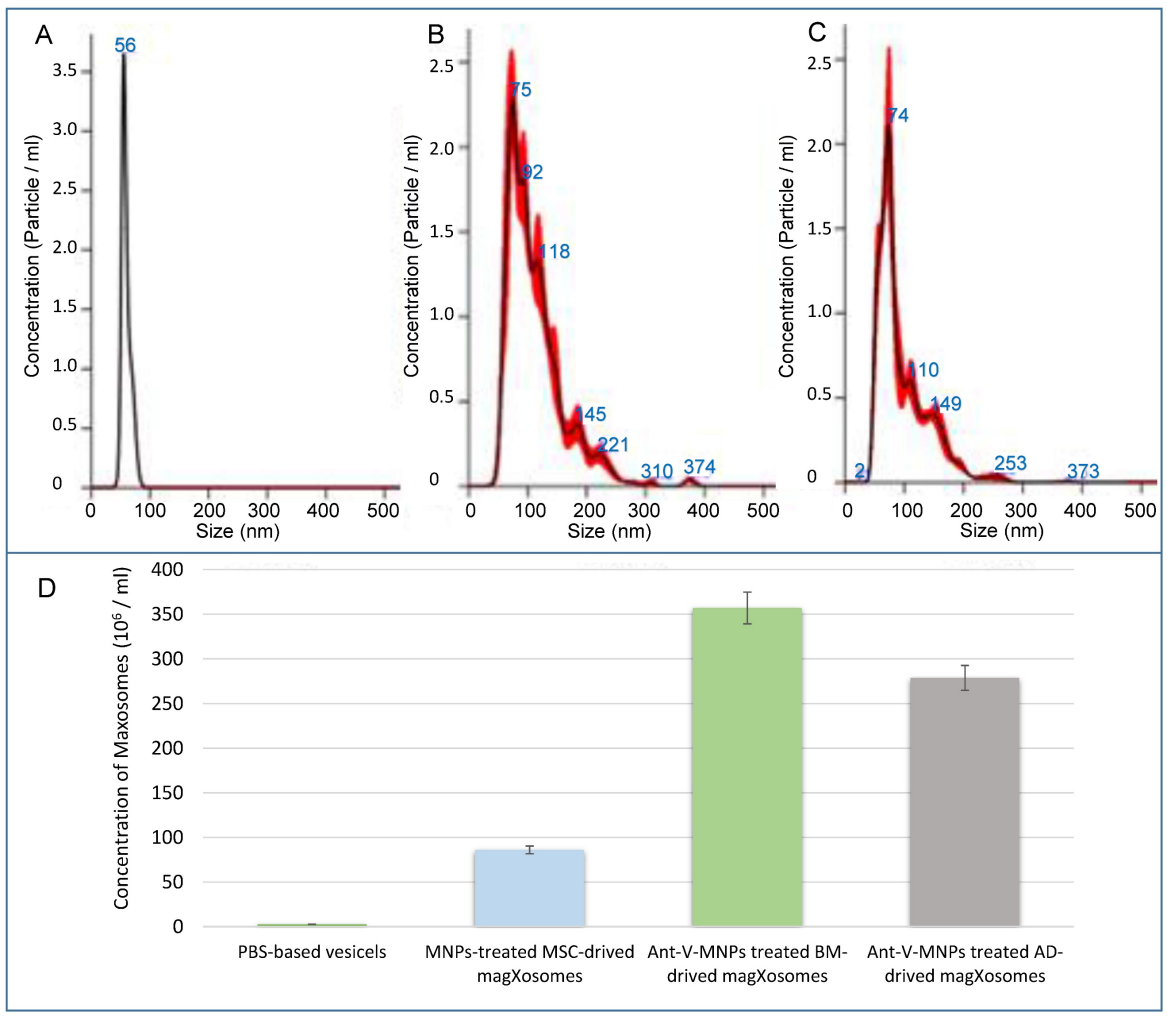
NTA analysis results; (A), (B), and (C) from left to right, PBS, BMSC-derived Magxosomes and ADSC-derived Magxosomes. (D) quantification of Magxosomes concentration. The bar graph shows the concentration of Magxosomes (10⁶/mL) isolated from BMSCs and ADSCs treated with Ant-V-MNPs and plain MNPs. The data indicate that the concentration of Magxosomes is highest for MSCs treated with Ant-V-MNPs, followed by MSC-derived Magxosomes treated with plain MNPs.

NTA analysis: In the following, features of Magxosomes, were evaluated with an NTA instrument. To this end, samples were diluted in filtered PBS to reach an appropriate concentration. By using the NTA device, particle size distribution and concentration of Magxosomes were recorded. All samples were characterized by a NanoSight NS500 instrument equipped with NTA 3.4 Build 3.4.003 version analytical software and a green laser. At least five 10-second videos were recorded per sample in light scatter mode with a camera level 16. Software settings for analysis were kept constant for all measurements (slider gain 295, detection threshold 5). The results are shown in Figure 3 and supplementary information Table S1. The acquired data showed that the size distribution of Magxosomes was approximately between 40 and 200 nm. In this regard, BMSC-derived Magxosomes were a little more abundant than ADSC-derived Magxosomes. Depending on the cell origin of Magxosomes, the concentration of Magxosomes varied slightly. According to the NTA-recorded curves, ADSC-derived Magxosomes exhibited a greater disparity in size distribution compared to BMSC-derived Magxosomes.

The recorded results regarding the concentration and size distribution of Magxosome particles confirmed that their abundance was consistent with previous studies 54, 55. The videos (supplementary information Table S1) showed the Brownian movement of individual particles of Magxosomes tracked by FTLA (fluorescently tagged lipid analogs) labeling, which could allow the visualization of individual tracks and their movement in real-time. It is also noteworthy that, for the investigation of Magxosomes stability, their zeta potential was measured using a Zetasizer. DLS analysis illustrated that Magxosome particles were stable in solution (Supplementary Information S1).

Overall, NTA analyzed data and Zetasizer measurement demonstrated that the size distribution, concentration, and stability of Magxosomes in solutions were appropriate and aligned with previous research 23. The final concentration of analyzed samples was 3.57 × 10⁸ and 2.79 × 10⁸ particles/mL for BMSC- and ADSC-derived Magxosomes, respectively. Notably, these particle counts per mL are attributed exclusively to the Magxosomes, which are predominantly smaller than 200 nm.

**Figure 4:**
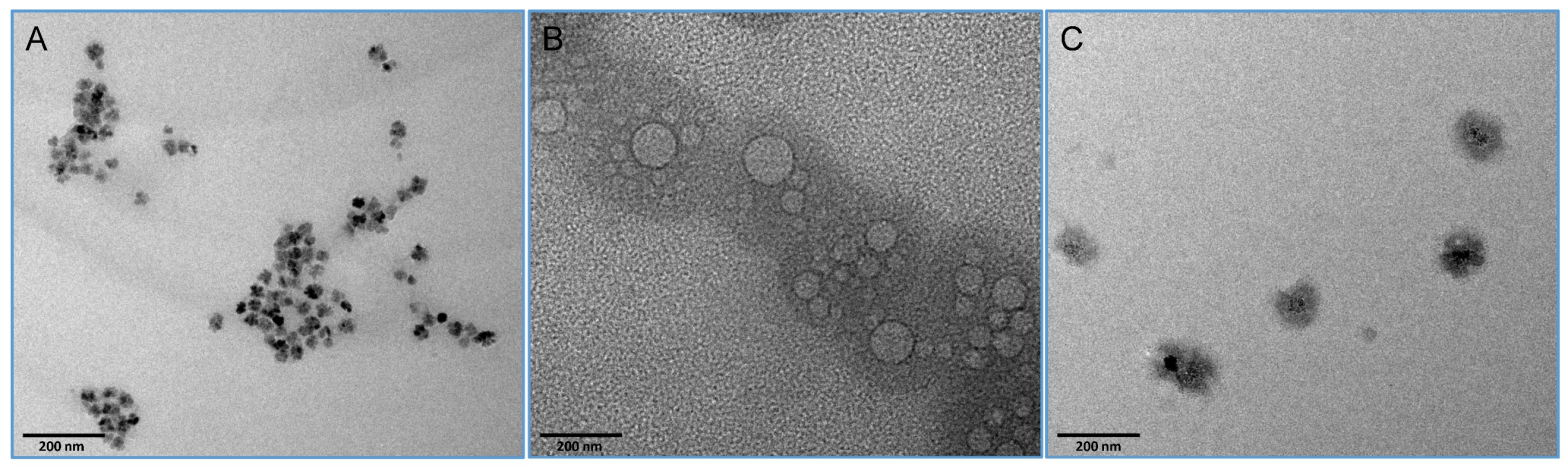
TEM images of (A) MNPs, (B) exosomes, and (C) Magxosomes. The scales bars and the quality (resolution) of TEM micrographs with mage J (http://imagej.org, Java 1.8.0_322 (64-bit), national institutes of health, USA).

TEM imaging: To determine the size and shape of Magxosomes, the TEM imaging analysis was applied, and the results are shown in Figure 4. The physical appearance of plain MNPs (Figure 4A), exosomes (Figure 4B), and Magxosomes (Figure 4C) are represented through TEM imaging. These data potentially approve the accuracy of produced Magxosomes. This is an appropriate method to advocate isolated exosomes condition. The size and shape of exosomes in their normal and magnetized states (Magxosomes) were evaluated. The micrographs of Magxosomes illustrated that their average sizes at dry state are almost around 100 nm. The appearances of MNPs, exosomes and Magxosomes are distinctive. In the case of Magxosomes, due to the presence of MNPs in their interior, the exosome bilayer membrane is not clearly illustrated. Indeed, exposing Magxosomes to electron beams resulted in an intense contrast between the MNPs (the inorganic part) and the exosome bilayer membrane (the organic part), which obscured the exosome structure 56. The difference between inorganic and organic materials is evident in TEM imaging due to their distinct atomic structures, which results in varying electron density and contrast 57. It is noteworthy that, measurement of samples was repeated twice to clearly visualize the differences. Regarding the differences between plain MNPs and Magxosomes, it was observed that plain MNPs exhibit a sharp contrast with an average size of approximately 15 nm. In contrast, the bilayer membrane of Magxosomes affects image clarity, leading to a reduction in resolution. The plain MNPs tend to form clusters, and their number is significantly higher than that of the Magxosomes. In contrast, Magxosomes are fewer in number and larger in size (around 100 nm), but their contrast intensity is lower.

ICP analysis: ICP-MS analysis was conducted to quantify the iron content of MNPs, mAbs-MNPs, and Magxosomes in blood samples. Iron concentration was measured using an ICP-MS instrument (7900, Agilent, Japan) following sample digestion with a Multiwave 7000 microwave system (Anton Paar, Australia). Samples were treated with nitric acid (HNO₃), sealed, and digested under controlled temperature and pressure to yield a homogeneous solution. While the microwave system enables efficient sample decomposition, it does not perform elemental analysis. The digested samples were then diluted with deionized water and analyzed by ICP-MS, which ionizes the sample in an argon plasma and detects elements based on their mass-to-charge ratio (m/z). The ICP-MS results confirmed the efficiency of the mAbs-MNPs conjugation process and supported the characterization of the isolated Magxosomes. The obtained results are presented in Supplementary Information Table S2. Based on the ICP data it was revealed that the iron concentration of plain MNPs was less than the recommended concentration by the manufacturer (5mg/ml). The concentration reported by the manufacturer includes the entire composition of the MNPs, comprising both the Fe_3_O_4_ core and the dextran shell. The ICP analysis in this research measured only the iron content. The empirical data from ICP analysis demonstrated that approximately 10 to 20% of the iron content in the samples was lost due to the conjugation process and washing steps. The iron concentration of the conjugated MNPs decreased compared to the initial plain MNPs. Regarding the Magxosomes, the ICP data are categorized into three parts: conjugated Ant-V-MNPs (the source of MNPs for MSC treatment), labeled cells (the origin cells of the Magxosomes), and isolated Magxosomes. The iron concentration in labeled MSCs and Magxosomes exhibits a consistent pattern of decreased iron content. Treatment of the MSC with Ant-V-MNPs showed that a notable part of the MNPs underwent uptake (supplementary information Table S2). ICP results showed that the concentrations of MNPs in BMSCs and ADSCs were nearly identical, suggesting a similar mechanism of MNP uptake in both cell types. However, data on the iron content of Magxosomes, combined with particle enumeration from NTA analysis, revealed some differences in the loading capacity of BMSC- and ADSC-derived exosomes. Specifically, the iron content in BMSC- and ADSC-derived Magxosomes was 0.31 µg/mL and 0.24 µg/mL, respectively. Although not statistically significant, this indicates a potential trend toward improved iron-loading efficiency in BMSC-derived Magxosomes (supplementary information, Table S3). However, to confirm this claim, additional experiments are necessary.

MPS signals and CTCs diagnosis: For the diagnosis of pancreatic cancer-associated CTCs in blood samples, a homemade MPS device recorded magnetic signals from two distinct diagnostic systems: mAbs-MNPs and Magxosomes nanoplatforms. The experiments were repeated three times, and for each experiment, 12 mice were sacrificed. The details of mouse body weight, tumor weight, and tumor size at the time of blood collection are presented in Table 2.

The blood samples were treated with mAbs-MNPs and Magxosomes platforms through different treatments (supplementary information Table S4). The recorded MPS signal indicated that Ant-V-MNPs is the most effective candidate among the various mAbs-MNPs samples for CTCs diagnosis. Indeed, blood samples treated with Ant-V-MNPs resulted in the highest signals in the MPS measurements. It evident that vimentin is an efficient biomarker in diagnosis of pancreatic cancer-associated CTCs. Vimentin expression increases during EMT, which in turn is a crucial process in CTCs biology.

**Figure 5:**
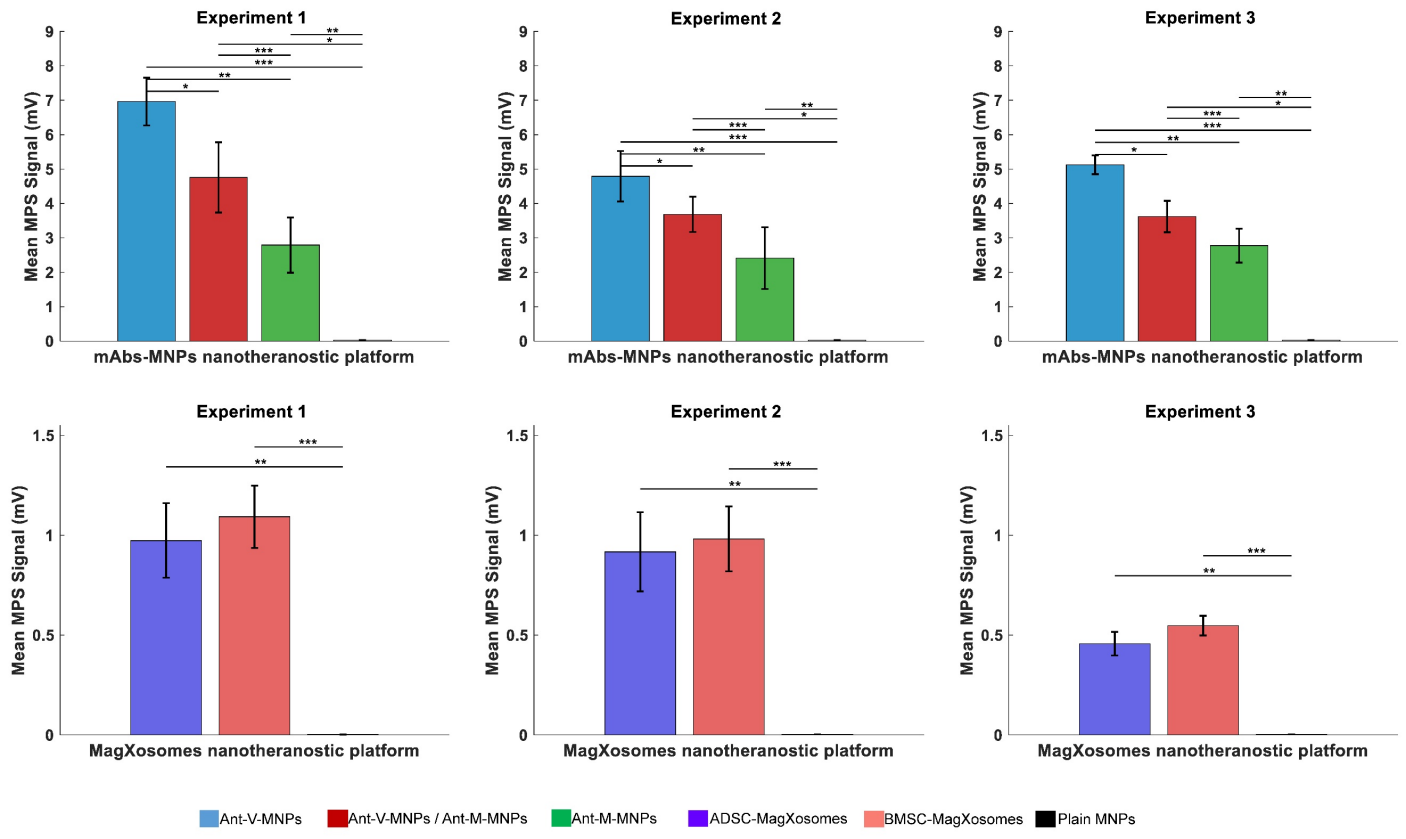
Mean and standard deviation (SD) values of MPS signals for mAbs-MNPs and Magxosome systems. (A), (B), and (C) display the results for mAbs-MNPs systems (Ant-V-MNPs, Ant-V-MNPs /Ant-M-MNPs, Ant-M-MNPs), while (D), (E), and (F) show the results for Magxosomes (BMSC- and ADSC-derived Magxosomes). Statistically significant differences are indicated based on post-hoc pairwise comparisons (* = p < 0.05, ** = p < 0.01, *** = p < 0.001). Error bars represent SD.

During EMT, MSCs attain the appropriate morphology to migrate in the extracellular environment and reside in tumor formation sites, in which interactions between epithelial cells and MSCs are involved 58. It has been demonstrated that vimentin is expressed not only in MSC but also in a variety of other cell types, such as pancreatic precursor cells, neuronal precursor cells, fibroblasts, and endothelial cells 16, 59. Nowadays, the highly conserved protein vimentin is considered the standard marker for EMT investigation. Heterogeneity property which is the manifest of CTCs is the outcome of EMT and generates a spectrum of intermediate phenotypes during the transition from epithelial to mesenchymal. Hence, the expression of vimentin can vary in this heterogeneous source of tumor cells 60-63. Previous studies demonstrated that vimentin protein is an appropriate candidate for the detection of pancreatic cancer blood samples' CTCs 64. The results of this study are consistent with previous research. The expression of the vimentin gene in cells undergoing EMT lead to the elevated vimentin levels in blood samples. Hence, blood samples treated with Ant-V-MNPs exhibited the highest recorded MPS signal values. The MPS recorded signals for each sample are presented in supplementary information Table S5, and the mean values for each system are plotted in Figure 5. Mesothelin (MSLN) is another chosen biomarker for CTCs detection. Even though the recorded signals of Ant-M-MNPs were lower than those of Ant-V-MNPs, it could still be considered as a proper candidate for CTC diagnosis. The MSLN is a glycoprotein, normally expressed on the surface of mesothelial cells covering the pleura, pericardium, and peritoneum. It has also a traceable amount of expression on the epithelial cell lining ovary, tunica vaginalis, rete testis, and fallopian tubes. However, overexpression of MSLN in cancers such as pancreatic, ovarian, lung adenocarcinoma, and gastric cancer has been reported 65-67. Studies illustrated that MSLN has high levels of expression in almost all pancreatic cancer cases, while normal pancreatic tissues have no expression 68, 69. Furthermore, highly expressed MSLN in solid tumors can be shed into the serum of patients and generate soluble MSLN-related protein (SMRP) which has the potential of target therapy 70.

Indeed, the MSLN precursor is cleaved into mature proteins, including megakaryocyte potentiating factor (MPF) and a cell surface glycoprotein known as MSLN. The MPF is secreted into the blood and used as a tumor marker 71-73. MSLN overexpression in pancreatic cancer increases MPF levels in the blood, enhancing the detection of CTCs expressing MSLN as a tumor marker specific to pancreatic cancer. A novel delivery system, called nanoimmunoliposome, was developed by conjugating anti-MSLN antibodies with MNPs and doxorubicin (DOX)-loaded PEGylated liposomes. This multifunctional nanoimmunoliposome proved effective as a diagnostic imaging agent for MRI monitoring and for targeted therapy of PDAC 74. In the present study, the MPS signals recorded from samples treated with Ant-M-MNPs confirm the effectiveness of MSLN in diagnosing PDAC-associated CTCs, consistent with previous studies.

The last recorded MPS signals belong to the samples treated with Ant-M-MNPs:Ant-V-MNPs, which in turn showed an intermediate pattern with a bias towards Ant-M-MNP signals. In fact, the Ant-M-MNPs/Ant-V-MNPs (50:50) is a mixed solution containing equal concentrations of Ant-M-MNPs and Ant-V-MNPs. However, the recorded signals illustrated that the Ant-M-MNPs were more efficient than the Ant-V-MNPs in the mixture. Several factors may contribute to this behavior. In this regard, the binding affinity and specificity of mAbs-MNPs to their targets could be factors to consider. Ant-M-MNPs demonstrated better binding affinity due to their target position (MSLN, a cell surface glycoprotein). As a result, they bind more strongly and potentially more rapidly to their target, enhancing their efficiency 75, 76. The MPS signal measurements in millivolts (mV) for plain MNPs used for conjugation (100 µg) and mAbs-MNPs platforms including Ant-V-MNPs, Ant-M-MNPs:Ant-V-MNPs, and Ant-M-MNPs, across three different experimental groups. The average MPS signal for each group is also provided. The other parameter which affects the behavior of conjugated MNPs is the kinetics of binding 77. Ant-M-MNPs appear to have a faster reaction rate, enabling them to bind to targets more quickly and thereby improving the overall system efficiency 78. In the same timeframe, Ant-M-MNPs bind more rapidly and achieve a higher rate of binding. Another possible reason is the occurrence of steric hindrance which interferes with the rate of results. Due to steric hindrance, Ant-M-MNPs under binding conditions can physically interfere with Ant-V-MNPs binding, thereby affecting the MPS signals 79. In this regard, the final configuration of Ant-M-MNPs in binding situations can influence the Ant-V-MNPs and restrict their navigation toward the targets 80. Differences in the properties of MSLN and vimentin may have varying effects on the magnetic properties of MNPs, influencing their behavior in a magnetic field and consequently affecting detection efficiency 81-83. It is also notable that Ant-M-MNPs and Ant-V-MNPs may differ in stability and tendency to aggregate, subsequently influencing their detection performance with MPS devices 84, 85. It is important to note that all the parameters mentioned above need to be investigated further to obtain a clear understanding of the main reasons for the effectiveness of Ant-M-MNPs in the results, which overshadow the effects of Ant-V-MNPs.

The targeted detection of pancreatic cancer-associated CTCs using engineered exosomes, referred to as Magxosomes, represents another novel approach in this research. The tumor-homing capability of MSCs is retained in the isolated Magxosomes, enabling them to track cancer cells 86-88. BMSC- and ADSC-derived Magxosomes have been investigated for their effectiveness in detecting CTCs. The multifaceted functionality of exosomes in cancer cell signaling and their capacity as noninvasive biomarkers evident them an excellent candidate in diagnosis and therapy 89. Related to pancreatic cancer, exosomes have significant clinical applications in diagnosis and therapy, for instance, blood-based exosomes have been analyzed through several different approaches and imaging techniques 90. In this study, MSC-derived Magxosomes were used as a theranostic platform to diagnose pancreatic cancer-associated CTCs in blood samples. The recorded MPS signals indicate that Magxosomes, whether BMSC- or ADSC-derived, exhibit high efficiency in diagnosing blood-based CTCs. A closer analysis of the recorded MPS signals, as shown in supplementary information Table S6 reveals that BMSC-derived Magxosomes demonstrate slightly better performance in detecting CTCs.

Statistical analysis of MPS signals for mAbs-MNPs platforms (Ant-V-MNPs, Ant-V-MNPs/Ant-M-MNPs, and Ant-M-MNPs) reveals variations in their performance. Two methods were applied: pairwise t-tests and one-way ANOVA, with significance set at p < 0.05. Through t-test analysis, the data reveals a significant difference, particularly in the case of Ant-V-MNPs compared to Ant-V-MNPs/Ant-M-MNPs, and Ant-M-MNPs. Furthermore, statistically significant differences were consistently observed between Ant-V/ Ant-M-MNPs and Ant-M-MNPs across all experiments Figure 5 (A-C), and supplementary information Table S8. Further validation of data through one-way ANOVA method, showing statistically significant differences in MPS signals between mAbs-MNPs platforms (p < 0.05). The bar graphs in Figure 1(A, B, and C), which depict the mean MPS signals and corresponding standard deviations, highlight the superior performance of Ant-V-MNPs, consistently yielding a stronger signal compared to Ant-V-MNPs/Ant-M-MNPs and Ant-M-MNPs. The error bars reflect the variability within the data for each system and their robust performance across all experiments.

Magxosomes, are also subjected to statistical analysis, and their performance proved to be interesting. In the first experiment, both BMSC- and ADSC-derived Magxosomes exhibited similar MPS signals (~1.09 mV and ~0.97 mV, respectively), with no statistically significant difference (p = 0.7). Data analysis from the second experiment showed a similar pattern in MPS signals (~0.98 mV and ~0.91 mV), also with no statistically significant difference (p = 0.77). In the third experiment, the difference in MPS signals became more apparent, with a statistically significant p-value (p < 0.05). In this regard, BMSC- and ADSC-derived Magxosomes exhibited MPS signals of approximately 0.55 mV and 0.46 mV, respectively, with a p-value of 0.056. However, since the results are not significantly different, additional empirical data are required to confirm the superior efficiency of BMSC-derived Magxosomes. Notably, NTA analysis data demonstrated that the loading capacity of BMSC-derived Magxosomes was slightly higher than that of ADSC-derived Magxosomes. Plotting the MPS signals in Figure 5 shows a similar pattern. However, this statistical analysis showed no significant difference. The results suggest that BMSC-derived Magxosomes may have enhanced performance compared to ADSC-derived Magxosomes, particularly in terms of loading capacity and detecting CTCs. Indeed, this is an estimate, and further empirical data are needed to conclusively demonstrate the superior efficiency of BMSC-derived Magxosomes. Statistical analysis results are presented in supplementary information Table S6.

**Figure 6:**
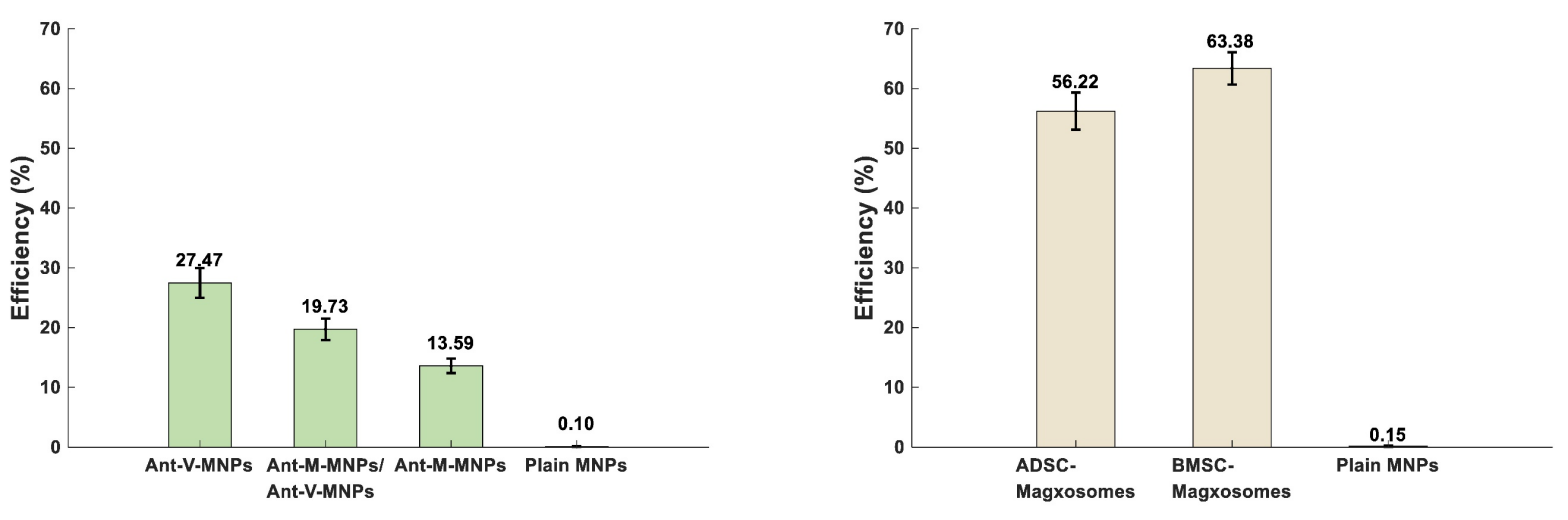
Performance efficiency (%) of developed nanotheranostic platforms for detection of CTCs. (A) shows the mAbs-MNPs systems, which include Ant-V-MNPs (27.47%), Ant-M-MNPs/Ant-V-MNPs (19.73%), and Ant-M-MNPs (13.59%). (B) shows the Magxosome systems, including AD-derived Magxosomes (56.22%) and BM-derived Magxosomes (63.38%). BM-derived Magxosomes exhibit the highest efficiency.

To determine the efficiency of the mAbs-MNPs and Magxosomes platforms in diagnosing CTCs, their performance was evaluated by analyzing the recorded MPS signals. As described in the methods, 50 µL of mAbs-MNPs (2 mg/mL) or Magxosomes (0.02 mg/mL) were mixed with individual blood samples. For each system, plain NPs were prepared: one sample contained 100 µg of MNPs, which served as the blank for the mAbs-MNPs platforms, while another sample contained 1 µg of MNPs designated for the Magxosomes. The MPS signals recorded from plain NPs served as references for calculating the efficiencies of the mAbs-MNPs and Magxosomes platforms. The formula for efficiency calculations is presented in supplementary information S1and the output is presented in supplementary information Table S7. The average efficiency of the mAbs-MNPs platforms across all three experiments indicates that Ant-V-MNPs have the highest efficiency at 27.47%, followed by Ant-M-MNPs/Ant-V-MNPs at 19.73%, and Ant-M-MNPs at 13.60% (Figure 6A). The calculation of Magxosomes efficiency (expressed as a percentage relative to the MPS signal of plain MNPs at a concentration of 1 µg) revealed that the average efficiency of BMSC-derived Magxosomes was 63.39%, while that of ADSC-derived Magxosomes was 56.23%. As shown in Figure 6B, BMSC-derived Magxosomes elicit a more pronounced response in MPS measurements, supporting their superiority over ADSC-derived Magxosomes as a platform for CTC detection.

Another important subject is the relative effectiveness of the nanotheranostic platforms in diagnosing CTCs. To this end, ICP data and MPS signals related to the platforms including Ant-V-MNPs and BMSC-derived Magxosomes, were selected as the most suitable candidates for diagnosis. ICP data were considered to reflect the quantities of the platforms, while MPS signals indicated their activities. The efficiency was calculated based on the ratio of activity to quantity. The result is represented in Table 3. The detailed ICP results and MPS signals for each platform are provided in supplementary information Table S8, while the formula for efficiency calculations is outlined in supplementary information S2.

The calculation estimated the diagnostic efficiency of Magxosomes for detecting blood-based CTCs to be 67.2% (8.2 / 0.122). This result suggests the superior performance of Magxosomes platforms in CTC detection. However, additional empirical data is necessary to substantiate this conclusion. The detection of MNP traces using the MPS device indicates the presence of CTCs in blood samples. The magnetic properties of MNPs are responsible for MPS tracing, while the presence of biomarkers accounts for their attachment to CTCs. This study developed a novel method for diagnosing pancreatic cancer-associated CTCs in blood samples, utilizing advanced homemade MPS technology combined with MNPs labeled with biological probes. It can be concluded that advanced MPS technology, combined with suitable biomarkers, provides quantified data that can be used for disease diagnosis. Overall, it can be stated that, as an interdisciplinary study, this research bridges engineering and biological sciences for applications in nanomedicine and molecular diagnostics. With further updates and optimization from both sides, MPS technology and biological markers, it has the potential to become more precise in detecting pancreatic cancer in its early stages.

## Conclusion

PDAC is an aggressive type of pancreatic cancer. Its high fatality rate positions it to become the second leading cause of cancer-related deaths in the near future. CTCs have the capacity to evade immune cells, persist in the circulatory system, and initiate metastasis to distant parts of the body. Due to the epithelial-mesenchymal transition (EMT) process, their appearance gradually changes, resulting in a heterogeneous population of cells. The transition from epithelial to mesenchymal cell shapes is associated with changes in the protein content of the CTCs. To maximize the detection of different phenotypes of CTCs, biomarkers offer a promising solution. This study demonstrates that combining advanced MPS technology with nanotheranostic platforms including mAbs-MNPs and Magxosomes, provides a novel approach for detecting pancreatic cancer-associated CTCs in blood samples. The results indicate that Ant-V-MNPs, which contain anti-vimentin antibodies, are the most effective among all variants of the mAbs-MNPs-based platforms. This approach enables efficient detection of pancreatic cancer-associated CTCs. The detection efficiency of the optimized platforms follows the order: Ant-V-MNPs > Ant-V-M-MNPs > Ant-M-MNPs. Regarding MSC-based Magxosomes, BM-Magxosomes appear to be slightly more effective than AD-Magxosomes. However, it is important to note that accurately determining the efficiency of MSC-based Magxosomes requires further experimentation. Another notable finding of this study is the superior performance of MSC-based Magxosomes compared to mAbs-MNPs-based platforms. The diagnosis of CTCs using Magxosomes proved significantly more efficient than with mAbs-MNPs-based platforms. The results demonstrate that Magxosomes outperform mAbs-MNPs-based platforms in detecting CTCs and may be recommended as a promising new biomarker for cancer diagnosis.

## Supplementary Material

Supplementary tables.

## Figures and Tables

**Table 1:** DLS data regarding particle size, zeta potential, and PDI of mAbs-MNPs samples

MNPs			Ant-M-MNPs			Ant-V-MNPs		
Average size	zeta potential	PDI	Average size	zeta potential	PDI	Average size	zeta potential	PDI
57.87	11.3	0.087	66.56	-4.43	0.234	61.48	-5.64	0.270
46.99	11.7	0.081	56.76	-5.98	0.228	63.3	-4.86	0.271
54.69	11.0	0.081	62.27	-5.72	0.243	81.26	-5.03	0.339

**Table 2:** Measurements of mouse body weight, tumor weight, and tumor size (longitudinal dimension) are presented. Weight is measured in grams (g), and tumor size is measured in millimeters (mm).

First experiment			Second experiment			Third experiment		
Mouse Weight (g)	Tumor Weight (g)	Tumor Size (mm)	Mouse Weight (g)	Tumor Weight (g)	Tumor Size (mm)	Mouse Weight (g)	Tumor Weight (g)	Tumor Size (mm)
20.9	1,39	17.23	18.5	0.94	10.05	19.3	0.61	6.87
20.1	0.96	11.75	19.3	0.57	4.50	16.7	0.62	6.92
18.4	0.68	7.89	20.7	1.06	13.58	18.4	0.81	9.37
21.1	0.83	10.99	19.6	0.78	8.31	19.3	0.76	7.89
17.3	0.66	6.87	18.8	0.80	8.85	17.6	0.97	11.9
19.1	0.71	6.92	20.4	0.86	9.79	18.9	0.83	9.15
20.6	0.81	9.96	19.5	0.88	9.41	20.1	1.26	15.25
19.7	0.79	8.15	20.0	0.75	8.31	19.0	1.16	13.58
19.9	0.60	5.66	18.2	0.67	6.69	18.7	1.0	12.04
20.6	0.98	11.37	19.9	0.56	4.94	20.3	0.94	10.80
18.5	0.91	9.41	18.4	0.68	6.82	21.3	1.11	12.22
21.4	0.81	8.82	18	0.69	7.50	19.8	1.31	14.47

**Table 3:** Calculations for the activity to quantity ratio

System	MPS Signal (V)	Quantity (ug)	Activity Ratio	Quantity Ratio	Activity-to-Quantity Ratio
Ant-V-MNPs	0.021181967	100	15.73	129.2	0.122
Magxosomes	0.001345969	0.774	0.06354	0.00774	8.2
