# Supplementary material for: Advanced Detection of Pancreatic Cancer Circulating Tumor Cells Using Biomarkers and Magnetic Particle Spectroscopy
Source: Nanotheranostics. 2025 Jun 12;9(2):171–85. doi: 10.7150/ntno.110074 (PMC12188535; doi:10.7150/ntno.110074)
Supplement: Supplementary file 1 — Supplementary tables. [file ntnov09p0171s1.pdf]

Supplementary Data

Table S1: Videos related to the Brownian motion of Magxosome particles, analyzed by NTA and zeta potential analyzed by DLS

Sample name	Brownian motion related Videos	zeta potential
PBS sample as the reference	PBS 2024-01-25 13-52-37-10sec.wmv	--
BM-derived Magxosomes particles	5 2024-01-25 15-30-02-10sec.wmv	-30.3
BM-derived Magxosomes particles	6 2024-01-25 15-43-34-10sec.wmv	-28.5
AD-derived Magxosomes particles	7 2024-01-25 15-56-03-10sec.wmv	-25.1
AD-derived Magxosomes particles	8 2024-01-25 16-10-32-10sec.wmv	-25.4

Table S2: The iron content of mAbs-MNPs and Magxosome samples was measured by ICP. Iron concentration is reported in mg/mL. The negative values in parentheses indicate the loss of MNPs during the washing steps following the conjugation process.

Sample name	Part-A (mAbs-MNPs platforms)			Blank	Part-B (Magxosomes samples)				
	Plain MNPs	Ant-V-MNPs	Ant-M-MNPs	water	MNPs used for treatment the MScs	Labeled-ADSC cell source	ADSC-derived exosomes	Labeled-BMSC cell source	BMSC-derived exosomes
Iron Concentration	3.15	2.69 (-14.6 %)	2.55 (-19.04 %)	0.073	3.315	1.024	0.784	1.019	0.841
	3.08	2.76 (-10.38 %)	2.56 (-16.8%)	0.0336	3.316	1.32	0.582	1.45	0.537
	4.185	3.54 (-15.4 %)	3.304 (-21.05 %)	7.33E-05	3.086	0.923	0.211	1.156	0.160
	4.342	3.48 (-19.8 %)	3.825 (-11.9 %)	0.0547	2.76	0.800	0.360	0.935	0.310

Table S3: MNPs loading efficiency in produced Magxosomes.

BMSC-based Magxosomes	ICP-data: Iron concentration (µg/ml)	NTA data: Magxosomes concentration (Particles /ml))	Concentration of iron (µg/ml) per 1×10^5 Magxosomes particles
	784	279644300	0.28
	582	142685714.3	0.40
	211	75772857.14	0.27
	360	133215714.3	0.27
Average	484	157829646.4	0.31
ADSC-based Magxosomes	841	357929143	0.23
	537	149137143	0.36
	160	144100000	0.11
	310	90095714.3	0.34
Average	462	185315500	0.24

Table S4: The experimental design including the specific treatments, volume of blood, and volume of specific treatment

Group	Treatment Description	Number of mice	Volume of blood (uL)	Volume of specific treatment (µL)
1	PBS (normal)	2	450	50
2	Ant-M-MNPs	2	450	50
3	Ant-M-MNPs/Ant-V-MNPs	2	450	50
4	Ant-V-MNPs	2	450	50
5	AD-derived Magxosomes	2	450	50
6	BM- derived Magxosomes	2	450	50

Table S5: MPS signals (mV) for different treatments of mAbs-MNPs and MagXosome systems across three individual experiments. The concentration of applied plain MNPs (100 µg) for MPS measurements of mAbs-MNPs samples was different from that of the plain MNPs (1 µg) used for MagXosome systems.

Experiment	Experimental Sample Repetition (R)	MPS Signals (mV)						
		Part A				Part B		
		Plain MNPs (100 ug)	mAbs-MNPs nanotheranostic platforms			Plain MNPs (1 ug)	Magxosomes	
			Ant-V-MNPs	Ant-M/Ant-V-MNPs	Ant-M-MNPs		AD-Magxosomes	BM-Magxosomes
1	R1	26.9545	7.945667	5.995467	3.5037	1.632564	1.0181	1.123267
	R2	27.0545	6.500367	4.881533	1.928867	1.602564	1.184933	1.277467
	R3	26.8545	6.960733	4.642833	3.442	1.572564	0.7341	0.900133
	R4	27.3215	6.436167	3.504467	2.293433	1.630126	0.9572	1.069
	Average	27.0463	6.960733	4.756075	2.792	1.609455	0.973583	1.092467
2	R1	20.5528	5.3181	4.061833	3.723133	1.642564	1.09181	1.0369
	R2	18.6217	5.187633	3.434567	1.983133	1.596026	1.084933	1.177467
	R3	18.4840	3.713333	3.082533	2.201833	1.572564	0.7341	0.800133
	R4	17.8083	4.9453	4.156767	1.734833	1.563026	0.7572	0.912227
	Average	18.8667	4.791092	3.683925	2.410733	1.593545	0.917011	0.981682
3	R1	14.0987	5.075678	4.003234	2.9123	0.955947	0.49246	0.606
	R2	17.0352	5.112856	3.504155	2.0802	0.849406	0.44032	0.545
	R3	15.9388	4.826124	3.951266	2.8618	0.912866	0.380933	0.486
	R4	18.4658	5.491653	3.025866	3.6432	0.890786	0.5133	0.553
	Average	16.3846	5.126578	3.62113	2.874375	0.902251	0.456753	0.5475

Table S6: Statistical analysis of p-values for mAbs-MNPs systems and Magxosomes. The table presents p-values calculated to compare various mAbs-MNP systems (Ant-V-MNP, Ant-V/Ant-M-MNP, and Ant-M-MNP) with each other, with the control group, and using ANOVA across three experiments. Additionally, the p-values for Magxosomes (AD-Magxosomes vs BM-Magxosomes and their comparisons with the control group) are shown for each experiment, along with ANOVA results for statistical significance.

p-Values were calculated to compare each mAbs-MNPs system with the others across all experiments							
Experiment	p_(Ant-V-MNP) vs p_(Ant-V/Ant-M-MNP)	p_(Ant-V-MNP) vs p_(Ant-M-MNP)	p_(Ant-V/Ant-M-MNP) vs p_(Ant-M-MNP)	Ant-V-MNPs Vs Control Group	Ant-V/Ant-M-MNPs Vs Control Group	Ant-M-MNPs Vs Control Group	ANOVA p_value
01	0.0119	0.0024	0.0232	0.0140	0.0124	0.0142	0.0018
02	0.0484	0.0062	0.0486	0.0091	0.0117	0.0099	0.0042
03	0.0013	0.0002	0.0450	0.0140	0.0124	0.0142	7.73e-05

p-Values of Magxosomes nanotheranostic platforms in comparison with other Magxosomes nanotheranostic platforms and control group across all experiments				
Experiment	p_ AD-Magxosomes vs p_ BM-Magxosomes	p_ AD-Magxosomes vs p_Control-group	p_ BM-Magxosomes vs p_Control-group	ANOVA_p_value
01	0.3654	0.0058	0.00089	0.0025
02	0.6317	0.0036	1.95e-05	0.0005
03	0.0562	1.35e-06	5.33e-07	1.78e-08

S1:

Using the average MPS signal values for each nanotheranostic platforms from Table S7 the efficiency of the nanotheranostic platforms will be calculated according to the following equation:

Efficiency = (MPS signal of the nanotheranostic platform / MPS signal of the Plain MNPs) x 100

Table S7: The relative effectiveness of the mAbs-MNPs and MagXosome systems' efficiencies in the diagnosis of CTCs was calculated as a percentage relative to the MPS signal of plain MNPs. Mean values of the MPS signal for each system from the Table S6 are used here for each experiment separately (mV) and the efficiencies are calculated.

Part A: Efficiency calculations for mAbs-MNPs systems											
Experiment # 1				Experiment # 2				Experiment # 3			
Plain (100 ug)	Ant-V-MNPs	Ant-M / Ant-V MNPs	Ant-M-MNPs	Plain (100 ug)	Ant-V-MNPs	Ant-M / Ant-V MNPs	Ant-M-MNPs	Plain (100 ug)	Ant-V-MNPs	Ant-M / Ant-V MNPs	Ant-M-MNPs
27.04	6.960	4.756	2.792	18.86	4.791	3.683	2.410	16.384	5.126	3.621	2.874

Efficiency calculated based on the MPS signal of plain MNPs which contains 100 ug iron content in each experiment					
Nanotheranostic platforms	Efficiency (%)	Nanotheranostic platforms	Efficiency (%)	Nanotheranostic platforms	Efficiency (%)
Ant-V-MNPs	25.73	Ant-V-MNPs	25.39	Ant-V-MNPs	31.28
Ant-M/Ant-V-MNPs	17.58	Ant-M/Ant-V-MNPs	19.52	Ant-M/Ant-V-MNPs	22.10
M-MNPs	10.46	M-MNPs	12.77	M-MNPs	17.54

Average efficiency calculated based on all three experiments (%)		
Ant-V-MNPs	Ant-M/Ant-V-MNPs	M-MNPs
27.47	19.73	13.59

Part B: Efficiency calculations for Magxosomes systems								
Plain (1 ug)	AD-Magxosomes	BM-Magxosomes	Plain 1 ug	AD-Magxosomes	BM-Magxosomes	Plain 1 ug	AD-Magxosomes	BM-Magxosomes
1.6094	0.9735	1.0924	1.5935	0.9170	0.9816	0.9022	0.4567	0.5475

Efficiency calculated based on the MPS signal of plain MNPs which contains 1 ug iron content in each experiment					
Nanotheranostic platforms	Efficiency (%)	Nanotheranostic platforms	Efficiency (%)	Nanotheranostic platforms	Efficiency (%)
AD-Magxosomes	60.49	AD-Magxosomes	57.54	AD-Magxosomes	50.62
BM-Magxosomes	67.87	BM-Magxosomes	61.60	BM-Magxosomes	60.68

Average efficiency calculated based on all three experiments (%)	
AD-Magxosomes	BM-Magxosomes
56.23	63.38

S2: As shown in the main text, calculations between the ICP data and MPS signals have been used to determine the relative effectiveness of the mAbs-MNPs and MagXosome systems in diagnosing CTCs, indicating their diagnostic efficiency. Specifically, the efficiency was calculated based on the ratio of activity to quantity. The results are presented in Tables S5. In this context, calculations for the efficiency of the nanotheranostic platforms are as follows:

1. MPS-based Signals: activity of the nanotheranostic platforms
2. ICP results gives the quantity of the nanotheranostic platforms
3. Activity-to-Quantity Ratio gives the efficiency of the nanotheranostic platforms

Table S8: The results of ICP (mg/L) and MPS (Volts) measurements across various nano systems, including Ant-M-MNPs, Ant-M-MNPs/Ant-V-MNPs, Ant-V-MNPs, AD-Magxosomes, and BM-Magxosomes, evaluated in three separate experiments alongside controls (100 ng plain NPs and untreated blood).

Experiment	Control Groups		Nanotheranostic platforms									
	Plain NPs (100 ng)	Un-treated blood	Ant-M-MNPs		Ant-M- MNPs / Ant-V-MNPs		Ant-V-MNPs		AD-Magxosomes		BM- Magxosomes	
	MPS (Volts)	MPS (Volts)	ICP (mg/L)	MPS (Volts)	ICP (mg/L)	MPS (Volts)	ICP (mg/L)	MPS (Volts)	ICP (mg/L)	MPS (Volts)	ICP (mg/L)	MPS (Volts)
1	0.02	0.0001	4.086	0.0049	3.2209	0.0075	2.7616	0.0045	0.0039	0.00024	0.00424	0.00055
2	0.017	0.0001	3.95	0.0052	2.8769	0.0013	2.853	0.0046	0.0041	0.00013	0.00452	0.00055
3	0.0187	0.0005	4.36	0.0143	2.9786	0.0160	2.9274	0.0258	0.0037	0.00016	0.00538	0.00033
Mean	0.0185	0.00023	4.132	0.0081	3.0254	0.0082	2.8473	0.0116	0.0039	0.00017	0.00471	0.00047
